# Source terms for benchmarking models of SARS-CoV-2 transmission via aerosols and droplets

**DOI:** 10.1098/rsos.212022

**Published:** 2022-05-04

**Authors:** Marc E. J. Stettler, Robert T. Nishida, Pedro M. de Oliveira, Léo C. C. Mesquita, Tyler J. Johnson, Edwin R. Galea, Angus Grandison, John Ewer, David Carruthers, David Sykes, Prashant Kumar, Eldad Avital, Asiri I. B. Obeysekara, Denis Doorly, Yannis Hardalupas, David C. Green, Simon Coldrick, Simon Parker, Adam M. Boies

**Affiliations:** ^1^ Department of Civil and Environmental Engineering, Imperial College London, London SW7 2AZ, UK; ^2^ Applied Modelling and Computation Group, Department of Earth Science and Engineering, Imperial College London, London SW7 2AZ, UK; ^3^ Department of Aeronautics, Imperial College London, London SW7 2AZ, UK; ^4^ Department of Mechanical Engineering, Imperial College London, London SW7 2AZ, UK; ^5^ Department of Mechanical Engineering, University of Alberta, Edmonton, Alberta, Canada T6G 2G8; ^6^ Department of Engineering, University of Cambridge, Cambridge CB2 1PZ, UK; ^7^ Fire Safety Engineering Group, University of Greenwich, London SE10 9LS, UK; ^8^ Cambridge Environmental Research Consultants Ltd, 3 Kings Parade, Cambridge CB2 1SJ, UK; ^9^ AEROS Consultancy, Glasgow G3 8SE, UK; ^10^ Global Centre for Clean Air Research (GCARE), Department of Civil and Environmental Engineering, Faculty of Engineering and Physical Sciences, University of Surrey, Guildford GU2 7XH, UK; ^11^ School of Engineering and Materials Science, Queen Mary University of London, London E1 4NS, UK; ^12^ MRC Centre for Environment and Health, Environmental Research Group, Imperial College London, Michael Uren Biomedical Engineering Hub, London, W12 OBZ, UK; ^13^ NIHR HPRU in Environmental Exposures and Health, Imperial College London, Michael Uren Biomedical Engineering Hub, London, W12 OBZ, UK; ^14^ Health and Safety Executive, Harpur Hill, Buxton, Derbyshire SK17 9JN UK; ^15^ Defence Science and Technology Laboratory, Porton Down, Salisbury SP4 0JQ, UK

**Keywords:** source terms, SARS-CoV-2, model benchmarking, exhaled aerosols, disease transmission

## Abstract

There is ongoing and rapid advancement in approaches to modelling the fate of exhaled particles in different environments relevant to disease transmission. It is important that models are verified by comparison with each other using a common set of input parameters to ensure that model differences can be interpreted in terms of model physics rather than unspecified differences in model input parameters. In this paper, we define parameters necessary for such benchmarking of models of airborne particles exhaled by humans and transported in the environment during breathing and speaking.

## 1. Introduction

Humans exhale particles made up primarily of respiratory fluid when breathing out, speaking, coughing, sneezing, singing and laughing and these particles may contain infectious pathogens [1,2]. The size of exhaled particles spans several orders of magnitude and particle diameters range between 0.01 and 1000 μm [3]. Historically, these particles have been classified into two categories by the infectious disease community: particles smaller than 5 μm in diameter are referred to as droplet nuclei or aerosols, whereas particles larger than 5 μm in diameter are classified as respiratory droplets [4,5]. This somewhat arbitrary size classification implicitly refers to the transmission modes/mechanisms, namely airborne or droplet transmission, respectively. However, the connection between particle diameter (droplets versus aerosols) and the description of transmission mode/mechanisms (droplet versus airborne transmission) can lead to misunderstanding. For example, it is untrue in general that particles with diameter greater than 5 μm fall quickly onto a surface close to their source since these particles, particularly those approximately 5–10 μm in diameter, can be advected with ventilation flows over longer distances and remain airborne for longer periods. Consequently, Prather *et al.* [6] recommend that aerosols and droplets are distinguished by a threshold of particle diameter of 100 μm, which more effectively separates their aerodynamic behaviour, ability to be inhaled and efficacy of interventions.

Particles are exhaled in a continuum of sizes and they rapidly change size depending on their environment, e.g. due to evaporation [7]. It is critical to understand the mechanisms of transport and deposition as a function of the size distribution of exhaled particles considering a range of external factors such as ventilation and air flows [8]. To that end, detailed experiments and models which accurately represent the relevant physics must be developed.

There is rapid advancement in approaches to modelling the fate of exhaled particles in different environments. These models have varying resolution and complexity in their representation of fluid flow and dispersion, and aerosol and droplet dynamics including evaporation, settling and transport [9–12].

As these modelling approaches evolve, it is essential to understand their robustness in representing the different physical processes. An important aspect of this is an objective inter-model comparison so that any differences in results can be attributed to alternative implementation of the physics or purposeful differences in modelled conditions. With this paper, we provide a consolidated set of parameters for exhalation of particles that can be used by a range of modelling approaches as the basis for model inter-comparison.

Droplets and aerosols produced by violent exhalation events, such as coughing and sneezing, have been investigated and reviewed by several studies [13–16]. Significant numbers of particles are also produced by breathing and speaking, activities which occur with greater frequency [17]. Under some circumstances, particularly in the case of presymptomatic or asymptomatic carriers who may not have symptoms of cough or sneezing, the cumulative amount of exhaled respiratory fluid as droplets and aerosols produced by high-frequency events such as breathing and speaking may be greater than that due to low-frequency intermittent events [18]. Furthermore, there remains uncertainty as to the importance of cough symptoms to transmission, with a recent study finding no association and that viral load, rather than symptoms, might be the predominant driver of transmission [19]. We therefore focus on defining parameters for breathing and speaking.

This paper has arisen from regular discussions between the authors, who have all engaged in review of existing and emergent evidence on respiratory disease transmission during the COVID-19 pandemic. It is not intended as a formal systematic review and therefore it is likely that there is some selection bias to our identification of literature. We only reviewed papers written in English and we did not apply pre-defined quality criteria to evaluate the strengths and weaknesses of different studies. Instead, this paper provides a careful examination of a selected literature that, in our view, provides a sound set of source terms for model benchmarking.

Details omitted from the main text are included in the electronic supplementary material where referenced.

## 2. Model parameters and conditions

The set of parameters which characterize exhalation of particles and environmental conditions relevant to particle transport are shown in table 1.

**Table 1.** Parameters for modelling exhalation of particles.

parameter	units	tidal breathing (nose)	tidal breathing (mouth)	speaking
exhalation (§2.1)				
area of opening	cm^2^	0.71	1.20	1.80
projection angle (side)	°	$\theta_{n,s}=60$	$\theta_{m,s}=0$	$\theta_{m,s}=0$
jet expansion angle (side)	°	$\phi_{m,s}=23$	$\phi_{m,s}=30$	$\phi_{m,s}=30$
projection angle (front)	°	$\theta_{n,f}=69$	—	—
jet expansion angle (front)	°	$\phi_{n,f}=21$	—	—
temperature	°C	34	34	34
relative humidity	%	100	100	100
source height	m	1.5	1.5	1.5
average flow rate	l min^−1^	10.6	10.6	12
exhaled particle size distribution (§2.2)				
mode 1: GMD_1_	μm	1.61	1.61	1.61
mode 1: GSD_1_	—	1.30	1.30	1.30
mode 1: *N*_1_	cm^−3^	0.0540	0.0540	0.0540
mode 2: GMD_2_	μm	—	—	2.40
mode 2: GSD_2_	—	—	—	1.66
mode 2: *N*_2_	cm^−3^	—	—	0.0684
mode 3: GMD_3_	μm	—	—	144.7
mode 3: GSD_3_	—	—	—	1.8
mode 3: *N*_3_	cm^−3^	—	—	0.00126
exhaled particle composition (§2.3)				
composition: salt, NaCl	g l^−1^	9	9	9
composition: protein, BSA	g l^−1^	3	3	3
composition: surfactant, DPPC	g l^−1^	0.5	0.5	0.5
molecular weight: NaCl	g mol^−1^	58.4	58.4	58.4
molecular weight: BSA	g mol^−1^	66 500	66 500	66 500
molecular weight: DPPC	g mol^−1^	734	734	734
density: NaCl	kg m^−3^	2160	2160	2160
density: BSA	kg m^−3^	1362	1362	1362
density: DPPC	kg m^−3^	1082	1082	1082
environmental conditions (§2.4)				
temperature	°C	20	20	20
pressure	atm	1	1	1
relative humidity	%	40	40	40

### 2.1. Exhalation

Gupta *et al.* [20] experimentally characterized various parameters associated with breathing and speaking; they measured gas flow rates, flow directions, and mouth and nose opening areas for 12 female and 13 male subjects. All subjects were healthy at the time of measurement and we note that there is a lack of literature on the potential effects of various symptoms of respiratory diseases on those parameters. The study documents significant variability among subjects and that flow rate is correlated to body surface area, which differs for males and females. The values listed in table 1 represent nominal values for three cases of tidal (restful) breathing through the nose or mouth, and speaking.

Different models may have different requirements or constraints with regards to their representation of breathing. Breathing could be modelled as an unsteady phenomenon, or it may be more simplistically modelled as a constant flow rate. We have determined a self-consistent set of parameters for both approaches by conserving the total volume of exhaled air (and therefore the total number of exhaled particles). However, we note that this leads to different flow velocities at the mouth or nose opening as exhalation only occurs for approximately half of the breathing period.

The breathing air flow rate (*Q*; [l s^−1^]) can be modelled by a sinusoidal function [20],

2.1 $$Q_{x}=a_{x}\;\sin(\beta_{x}t),$$

where *t* is time (s), the subscript *x* indicates either inhalation (in) or exhalation (out), *β*_*x*_ = *π* RF_*x*_/30 is a function of the respiratory frequency (RF; [min^−1^]), and *a*_*x*_ = *β*_*x*_ TV/2. The RF for inhalation (RF_in_) and exhalation ($RF_{\mathrm{out}}$) are given as functions of body height (*H*; [cm]) and body mass (*W*; [kg]) by eqns (7)–(10) in Gupta *et al.* [20] and shown in electronic supplementary material, S1. The tidal volume (TV; [l]) is given as

2.2 $$\mathrm{TV}=\frac{\mathrm{MV}({\mathrm{RF}}_{\mathrm{out}}+{\mathrm{RF}}_{\mathrm{in}})}{2{\mathrm{RF}}_{\mathrm{out}}{\mathrm{RF}}_{\mathrm{in}}},$$

where the minute volume (MV; [l min^−1^]) is the volume of air exhaled in 1 min (sometimes also referred to as the minute ventilation). The derivation of equation (2.2) is shown in electronic supplementary material, S1. MV is correlated with the body surface area (BSA; [m^2^]) by MV = *c* × BSA. The constant *c* ([l min^−1^ m^−2^]) is 5.225 and 4.634 for males and females, respectively [20]. The BSA can be estimated according to Gehan & George [21],

2.3 $$\mathrm{BSA}=0.02350H^{0.42246}W^{0.51456},$$

where *H* is height in centimetres and *W* is body mass in kilograms. Considering the average British male and female, who are 175.3 and 161.6 cm tall and weigh 83.6 and 70.2 kg, respectively [22], we obtain $a_{\mathrm{in}}=0.5956$, $\beta_{\mathrm{in}}=2.0629$, $a_{\mathrm{out}}=0.5215$ and $\beta_{\mathrm{out}}=1.8061$ for males and $a_{\mathrm{in}}=0.0.4794$, $\beta_{\mathrm{in}}=1.6722$, $a_{\mathrm{out}}=0.3991$ and $\beta_{\mathrm{out}}=1.3922$ for females. Thus, the breathing flow rate ($Q_{\mathrm{breathing}}$; [l s^−1^]) over the cycle of inhalation and exhalation can be represented by a piecewise sinusoidal function with a period of *π*/*β*_in_ + *π*/*β*_out_,

2.4 $$Q_{\mathrm{breathing}}=\left\{ \begin{array}{cc} -a_{\mathrm{in}}\;\sin(\beta_{\mathrm{in}}t)\; & 0\leq t\leq \pi /\beta_{\mathrm{in}}\;\\ a_{\mathrm{out}}\;\sin\left(\beta_{\mathrm{out}}\left(t-\frac{\pi }{\beta_{\mathrm{in}}}\right)\right)\; & \pi /\beta_{\mathrm{in}}<t\leq \left(\frac{\pi }{\beta_{\mathrm{in}}}+\frac{\pi }{\beta_{\mathrm{out}}}\right).\; \end{array}\right.$$

**Figure 1.**
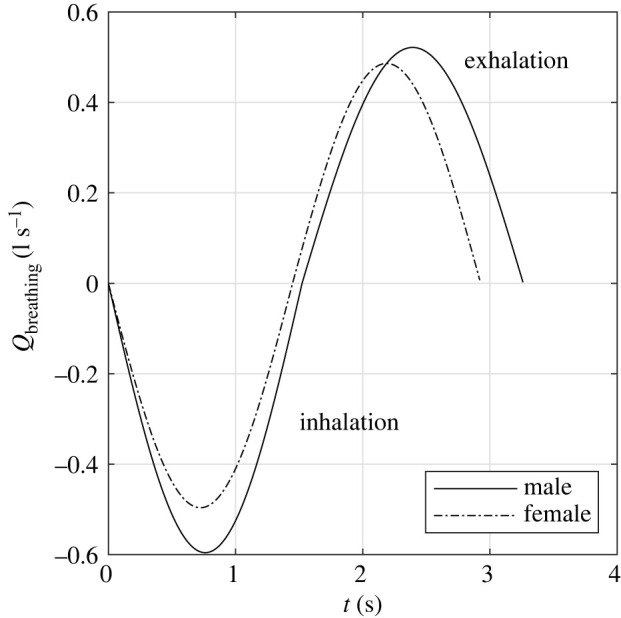
Graphical representation of the breathing flow rate.

A graphical representation of $Q_{\mathrm{breathing}}$ for the average British male and female are shown in figure 1.

Alternatively, the volumetric production of exhaled air may be modelled as a steady process, in which case the average flow rate is obtained by dividing the total exhaled volume by the breathing period. Using the same values of $a_{\mathrm{out}}$ and $\beta_{\mathrm{out}}$, we obtain an average exhalation flow rate of 10.6 l min^−1^ (0.177 l s^−1^) and 8.3 l min^−1^ (0.139 l s^−1^) for the average male and female, respectively. These values are close to those recommended for representing breathing rates in risk assessments [23].

For speaking, the breathing pattern is not sinusoidal and varies significantly with the vocalization. A nominal average exhalation flow rate is 12 l min^−1^ (0.2 l s^−1^) for vocalizing passages of text [20]. While this is adequate for model comparison, we encourage readers to study the original reference for values that may be more representative of specific cases and to other literature that has measured the spread of exhalation flow rates for different individuals and vocalizations, which suggest that exhalation flow rates during singing are similar to those during speaking [24].

**Figure 2.**
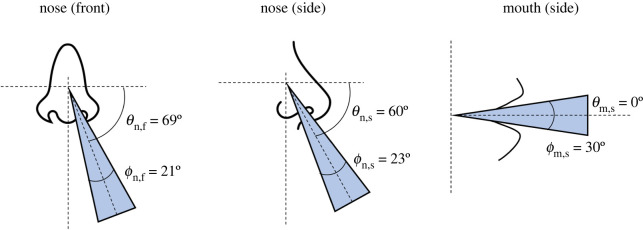
Graphical representation of jet projection (*θ*) and spreading (*ϕ*) angles.

For the purposes of model comparison we include nominal average exhalation flows, assuming steady flow and the average British male, in table 1. Nominal projection and spreading angles of the jets of exhaled air from the nose and mouth are also taken from Gupta *et al.* [20] and they are shown graphically in figure 2. For nose breathing, we suggest that it is appropriate to assume that the exhaled air flow is split equally between two nostrils. However, we note that there is normally asymmetry in these flows due to anatomical, physiological and disease factors that shift and alternate the asymmetry over time [25].

### 2.2. Exhaled particle size distribution

**Figure 3.**
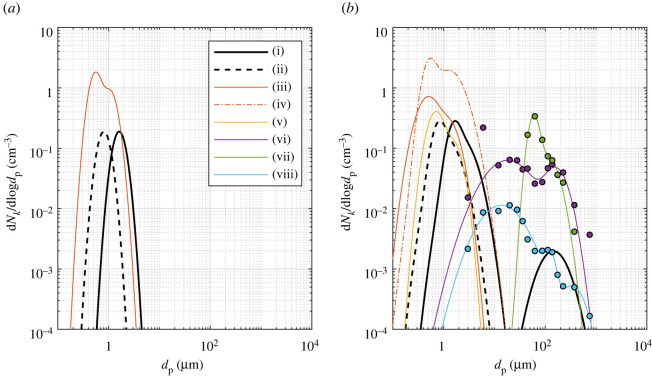
Exhaled particle size distributions resulting from (*a*) breathing and (*b*) speaking from (i) Johnson *et al.* [27] corrected for particle shrinkage and representing the PSD at the mouth (*BLO model*), (ii) Johnson *et al.* [27] not corrected for particle shrinkage, (iii) Gregson *et al.* [32] (70–80 dBA in (*b*)), (iv) Gregson *et al.* [32] (90–100 dBA in (*b*)), (v) Asadi *et al.* [31] (electronic supplementary material, figure S10), (vi) Chao *et al.* [29], (vii) Xie *et al.* [28] and (viii) Duguid [26]. Parameters of lognormal distributions and further information on the sources of data are included in electronic supplementary material, S2.

The earliest measurements of exhaled particle sizes relied on the microscopic analysis of droplet marks on slides placed in front of the mouth [26] and these techniques are still used to estimate exhaled particle counts for particle diameters greater than 10 μm [27,28]. Optical techniques have also been used to measure exhaled particles with diameters greater than 1 μm [29,30]. In studies using the droplet deposition and microscopy methods, it is common for the total number of particles counted within different size ranges to be reported, rather than the concentration of particles in exhaled breath and corrections are typically applied to the measured particle size distribution to account for artefacts such as evaporation or spreading of the droplets on the surface of the slide. To measure particles of diameter less than 10 μm, a number of studies have relied upon measurements using the aerodynamic particle sizer (APS, Model 3321, TSI Inc.), which has a manufacturer-specified particle aerodynamic diameter detection range of 0.5 to 20 μm [27,30–32]. These measurements are affected by the evaporation of water from the exhaled particles as they are expelled from the high humidity conditions in the body to the lower humidity of the experimental environment. The authors of these studies acknowledge that this process of droplet drying happens in the timescale of approximately 1 s [7] and that the measured size distribution is representative of the equilibrium size distribution. Johnson *et al.* [27] applied a correction to account for the shrinkage of particles due to evaporation, whereas other studies have chosen not to correct for this process. Another important distinction between studies measuring particles in this size range is studies have either sampled a small fraction of the exhaled air flow [31,32] or have sampled the plume of exhaled air and corrected the measured concentration for plume dilution, as measured using a trace gas (e.g. water) [27]. A comparison of particle size distributions from different studies is shown in figure 3 and details of the source of data for this plot can be found in electronic supplementary material, S2.

Johnson *et al.* [27] reported that particles generated from breathing, speaking and coughing were present in a range of sizes, represented by distinct modes of a frequency distribution of particle diameters that spans from 0.1 to 1000 μm. They propose the *BLO model* for the size distribution of particles measured: bronchiolar (*B*), laryngeal (*L*) and oral (*O*) to represent the different locations in the airways believed to be the source of the aerosols.

A recent publication showed that patients admitted to hospital with COVID-19 exhaled similar aerosol size distributions to healthy patients when breathing, speaking and coughing [33]. We also note that the studies that we have reviewed either did not mention differences in particle size distributions for male and female subjects [27,29], or could not find any statistically significant difference [28,31,32].

#### 2.2.1. Bronchiolar and laryngeal particles

Particle diameters from the first two modes, bronchiolar and laryngeal, were found to range from at least 0.5 to 5 μm, both with median diameters of order 1 μm using on-line measurement techniques using the APS and after correction for evaporation by assuming a shrinkage factor of 0.5 [27]. The evaporation-corrected size distribution represents the initial particle size distribution at the mouth and can be compared to the uncorrected equilibrium size distribution in figure 3. Recently, Asadi *et al.* [31] and Gregson *et al.* [32] reported equilibrium particle size distributions for breathing and speaking. For speaking, both studies report significant variability with respect to the loudness of vocalization and among individuals. As shown in figure 3, these two studies are in good agreement with the uncorrected size distribution from Johnson *et al.* [27] with respect to median diameters. However, the three studies span approximately an order of magnitude in concentration and the size distributions from Asadi *et al.* [31] and Gregson *et al.* [32] appear to have a larger spread (i.e. geometric standard deviation). The difference in concentration between studies is likely within the range of variation due to vocalization, loudness and individual variability; however, it is possible that sampling and data processing differences may also contribute.

While we focus here on breathing and speaking, we acknowledge that there are recent studies reporting particle size distributions for singing [30,32]. While singing is found to increase the number concentration of exhaled particles relative to speaking, the increase is small relative to the changes associated with increased loudness [32].

#### 2.2.2. Oral particles

Johnson *et al.* [27] reported that the oral mode of particles measured during speaking were larger than 10 μm in diameter and all contained food-dyed saliva, demonstrating that those particles originated from the mouth. This observation of the presence of food-dye is in agreement with Duguid [26] and Xie *et al.* [28], and data from these two studies are also shown in figure 3. We have also included the optical measurements from Chao *et al.* [29] in figure 3 and it is evident that there is significant variation in the magnitude, mode and spread of size distributions for oral particles. These differences may be attributed to differences in measurement techniques, vocalizations and variability among individuals. It is beyond the scope of this paper to review these differences in detail, however, we note the need for further studies that compare different measurement approaches, for example, by conducting simultaneous measurements using different techniques of the same exhaled aerosol, and the interested reader is referred to the following additional references [34–37]. We recommend that the oral particle size distribution for speaking is treated as more uncertain than the bronchiolar and laryngeal modes. The parameters for the size distributions from different studies are included in electronic supplementary material, S2 to enable model sensitivity studies. There is limited evidence of exhaled aerosols with diameters greater than 10 μm as a result of singing.

#### 2.2.3. Parameter specification

The discussion above indicates that there is significant variability in exhaled particle concentration and size distribution due to respiratory activity and individual variability. For the purposes of model comparison, we adopt the *BLO model* [27], corrected to represent the particle size distribution at the mouth (series (i) in figure 3), as the basis of the terms included in table 1. We note that this particle size distribution is representative of the mean for the group of healthy volunteers in that study and is therefore not predictive of a single person as inter- and intra-person variability is of the order of measured concentration itself or greater [27].

**Figure 4.**
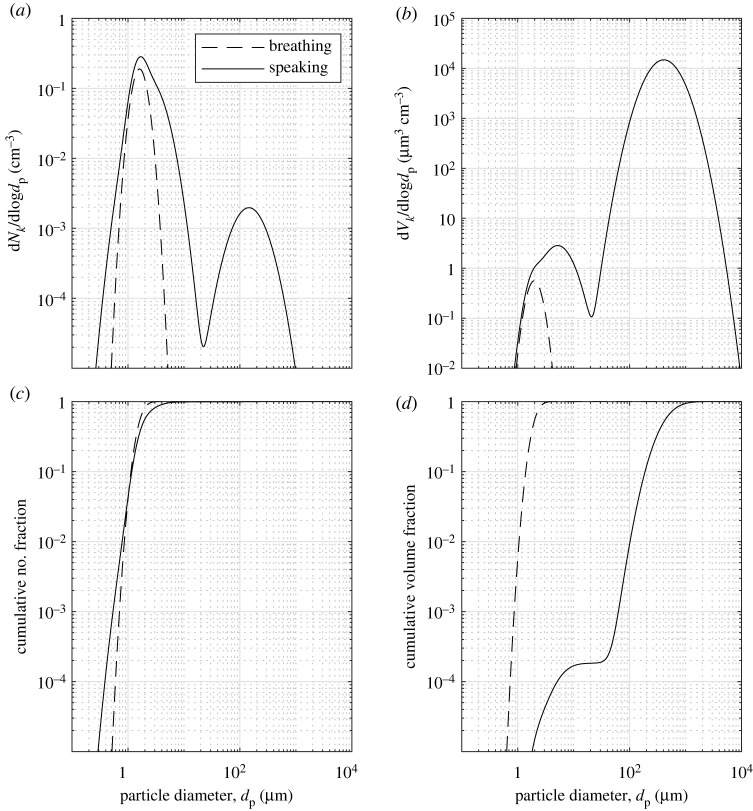
(*a*) Number and (*b*) volume weighted particle size distributions, and cumulative fractions of (*c*) particle number and (*d*) volume as a function of particle diameter for breathing and speaking.

For breathing, only the *B* mode is included. For speaking, the size distribution of exhaled particles is the sum of the three *B*, *L* and *O* lognormal distribution modes [27],

2.5 $$\frac{dN_{k}}{d\;\log\;d_{\;p}}=\ln(10)\sum_{i=1}^{3}\left[\left(\frac{N_{i}}{\sqrt{2\pi }\;\ln({\mathrm{GSD}}_{i})}\right)\exp\left(-\frac{{\left(\ln\;d_{\;p}-\ln\;{\mathrm{GMD}}_{i}\right)}^{2}}{2{\left(\ln\;{\mathrm{GSD}}_{i}\right)}^{2}}\right)\right],$$

where $d_{\;p}$ is the particle diameter (μm), *N*_*i*_ is the total number concentration (cm^−3^) of each mode *i*, GMD_*i*_ is the geometric mean diameter (μm) of each mode *i* and GSD_*i*_ is the geometric standard deviation of each mode *i*. Each mode may be characterized by only three parameters: GSD_*i*_, GMD_*i*_ and *N*_*i,*_ as given in table 1. The particle size distribution for breathing and speaking is shown in figure 4*a*.

The notation $dN_{k}/d\;\log\;d_{p}$ represents the number concentration in each bin of particle diameters (d*N*_*k*_) normalized by a bin width that is constant in log space, i.e. $d\;\log\;d_{p}=\log(d_{p,k+1}/d_{p,k}$), where *k* represents a discretization of the $d_{p}$ space. Note that log here refers to the base 10 logarithm and equation (2.5) is preserved from Johnson *et al.* [27]. Therefore, the absolute number concentration of particles of a given bin of particle diameters (d*N*_*k*_; [cm^−3^]) is calculated as

2.6 $$dN_{k}=\frac{dN_{k}}{d\;\log\;d_{\;p}}d\;\log\;d_{\;p}.$$

In the context of exhalation, it is important to consider both number and volume of exhaled particles. The volume of particles of a given diameter represented as a concentration [μm^3^ cm^−3^], assuming all particles are spherical, is given by

2.7 $$dV_{k}=\frac{dV_{k}}{d\;\log\;d_{\;p}}d\;\log\;d_{\;p}=dN_{k}\left(\frac{\pi d_{p,k}^{3}}{6}\right).$$

The volume weighted particle size distribution and cumulative number and volume fractions are shown in figure 4*b*–*d* for breathing and speaking. The total number concentration, *N*, of particles is 0.054 cm^−3^ for breathing and 0.1237 cm^−3^ for speaking and the total volume concentration, *V*, is 0.1608 μm^3^ cm^−3^ for breathing and 9.4637 × 10^3^ μm^3^ cm^−3^ for speaking, summarized in table 2.

**Table 2.** Estimates of concentrations and emission rates of particles.

parameter	units	breathing	speaking
nominal average flow rate: $\bar{Q}$	cm^3^ s^−1^	176	200
exhaled number concentration: *N*	cm^−3^	0.054	0.124
exhaled volume concentration: *V*	μm^3^ cm^−3^	0.161	9.46 × 10^3^
	(ml cm^−3^)	(1.61 × 10^−13^)	(9.46 × 10^−9^)
avg. number emission rate: ${\bar{E}}_{N}$	s^−1^	9.50	24.7
avg. volume emission rate: ${\bar{E}}_{V}$	μm^3^ s^−1^	28.3	1.89 × 10^6^
	(ml s^−1^)	(2.83 × 10^−11^)	(1.89 × 10^−6^)

The release rate of particle number (*E*_*N*,*k*_; [s^−1^]) or volume (*E*_*V*,*k*_; [μm^3^ s^−1^]) for a given particle diameter is calculated as the product of the particle number or volume concentration and the exhaled flow rate, i.e.

2.8 $$E_{N,k}=dN_{k}\;\max[0,Q_{\mathrm{breathing}}]$$

and

2.9 $$E_{V,k}=dV_{k}\;\max[0,Q_{\mathrm{breathing}}].$$

For example, considering a nominal average flow rate of 12 l min^−1^ (200 cm^3^ s^−1^) for vocalizing passages of text [20] and exhaled number, *N*, and volume, *V*, concentrations for speaking yields emission rates of ${\bar{E}}_{N}=24.7\;s^{-1}$ or volume ${\bar{E}}_{V}=1.89\times 10^{6}\;\mu m^{3}\;s^{-1}$ (1.89 × 10^−6^ ml s^−1^). Estimates of particle emission rates during breathing and speaking are summarized in table 2, highlighting that speaking produces an estimated 6.7 × 10^4^ times larger volume of fluid than breathing alone, primarily from the oral mode of droplets (typically larger than 10–50 μm) originating from the mouth.

### 2.3. Exhaled particle composition

Exhaled particles are multi-component droplets comprising water, salts, proteins and surfactants [38–40]. Once exhaled from the nose or mouth, these particles are exposed to a rapidly changing relative humidity (RH) within the exhaled breath from approximately 100% to ambient conditions. The combination of the droplet composition and ambient temperature and RH will influence the evaporation rate and therefore affect settling times of a single respiratory droplet [7,41,42]. As a multi-component droplet with non-volatile solutes evaporates, the evaporation rate may change throughout the process due to an increase in concentration of solutes in the liquid, as well as other physico-chemical transformations [38]. The resulting size of the droplet, represented by a characteristic diameter, after it has come into equilibrium with the ambient conditions, not only determines its settling time [11,28,41,42] but also its fate in the respiratory system should it be inhaled by an individual [5,43,44]. When considering the whole range of sizes found in respiratory releases (figure 4), the combined effect of RH and composition may result in up to an order-of-magnitude difference in the total amount of suspended mass of a droplet cloud of different compositions [11].

For the purposes of model comparison, we suggest a four-component droplet composition consisting of 9 mg ml^−1^ of NaCl, 3 mg ml^−1^ of protein (bovine serum albumin, BSA), and 0.5 mg ml^−1^ of surfactant (dipalmitoylphosphatidylcholine, DPPC) in water. This protein concentration is representative of the composition of nasal surface airway fluid [45] and this simplified composition is comparable to concentrations in simulated lung fluid [42,46,47], and has been used in a previous modelling study [11]. The concentration of each component, together with their respective molecular weight and density [48–51], are given in table 1. Properties of water for modelling the dynamics of particles including evaporation are readily available from e.g. Green & Perry [52]. The three other components (i.e. NaCl, protein and surfactant) are not volatile at typical ambient conditions due to their significantly higher molecular weights and melting points [53,54]. We note that when modelling the dispersal of virus within respiratory fluid (cf. §3), the contribution of virus particles to the bulk composition of the particle is negligible for typical viral loads.

### 2.4. Environmental conditions

The temperature and RH of ambient air significantly affects the fate of exhaled particles, in terms of the rate of evaporation of water from droplets [11,55], and, while not explicitly relevant to defining source terms, the inactivation rates of enveloped viruses [41,56,57].

Guidelines for different indoor environments are published by various regulatory bodies. For example, guidelines for ward spaces and intensive care units in hospitals, set design air temperature and RH ranges between 20–24°C and 30–60%, respectively [58] and similar guidelines exist for schools in different countries [59–61].

Empirical studies on indoor temperature and RH in different environments suggest that these can vary with the seasons and that there is variability between buildings used for the same purpose. In three hospitals in the USA, temperatures were measured to within the recommended range of 20–24°C; however, RH was consistently below 40% in all locations [62]. In two hospitals in France, average temperatures and humidity ranged from 19–27°C and 16–70%, respectively, across all seasons [63]. For low-income households in the UK in winter, median indoor temperature and RH were found to be 19°C (14−23°C, 5th to 95th centile range) and 43% (32–60%) in living rooms, respectively [64], with significant variability by season and dwelling type [65]. For dwellings in the USA, median indoor temperature and RH were found to be 20°C (18−27°C, range) and 48% (23–71%), with seasonal variations in RH [66]. In industrial settings, there may be indoor conditions that are specific to the activity and setting, e.g. meat processing [67], and standards and outdoor conditions will have an effect in different climatic regions [68,69].

The empirical evidence suggests that temperature and RH span the range of 20–24°C and 30–60%, respectively. For model comparison, we propose an ambient temperature of 20°C and an RH of 40%, which are included in table 1. Given the importance of these parameters, we would encourage researchers to present results for the ranges of 15–30°C and 30–60%, as a minimum.

## 3. Pathogens in exhaled particles

There is limited evidence for the amounts of pathogens possibly contained in particles exhaled by different respiratory activities and significant variability among different types of pathogens, therefore, we do not include values for concentrations of pathogens in our set of parameters for exhaled particles. However, considering the recent focus on modelling transmission of SARS-CoV-2, below we discuss the data for SARS-CoV-2 to help readers make more informed judgements on appropriate viral load values for their modelling efforts.

### 3.1. Prevalence of SARS-CoV-2 in indoor air

At the time of writing, the viral load and infectivity of SARS-CoV-2 in exhaled particles of different sizes has not been well established [70]. Gene copies^1^ of SARS-CoV-2 ribonucleic acid (RNA) have been detected by polymerase chain reaction (PCR) analyses of samples of indoor air gathered in a range of (mostly clinical) settings [71], including in aerosols smaller than 5 μm [72–74]. In indoor air, the concentrations of SARS-CoV-2 RNA reported in particles smaller than 5 μm are of order 1 × 10^−5^ [72] to 1 × 10^−3^ [73–75] gene copies per cm^3^ of sampled air.

Importantly, modellers must note that the number of SARS-CoV-2 gene copies detected by PCR quantifies sub-sections of the viral RNA sequence and is therefore not equal to the number of infectious viruses present. However, based on a range of clinical samples (e.g. nasopharyngeal swabs), the likelihood of detecting infectious SARS-CoV-2 by viral culture methods is correlated with number of gene copies reported where RNA viral loads greater than 10^5^−10^6^ gene copies/ml (corresponding to *Ct* < ∼24−25) and higher are typically required to demonstrate infectivity of a clinical sample containing SARS-CoV-2 [76–83]. To date, cycle thresholds for the air samples that detect SARS-CoV-2 RNA are very often greater than 30 and even greater than 35, implying air samples are often not likely to culture [71]. Of attempts to demonstrate the infectivity of SARS-CoV-2 suspended in field samples of indoor air by viral culture methods [75,84–87], there has been limited evidence of viral replication or cytopathic effects (CPE) [87–89].

Plaque assay, a cell culture method, when performed on samples with higher viral loads than typically found in air samples (e.g. nasopharyngeal swabs), enables quantification of the number of infectious viruses capable of forming plaques in a cell monolayer, called plaque-forming units (PFU). These PFUs may be used in dose–response models to estimate infection risk in humans (as done for SARS, for example [90]). Syrian hamsters inoculated by the intranasal route were infected with a dose of as low as 14 PFUs and the minimum infectious dose may be lower in humans [82]. Since there is insufficient data on the possible load of infectious viruses in air samples, it is appropriate to estimate a possible range based on the number of gene copies detected. A ratio of RNA gene copies (N Gene) to PFUs of approximately 160 000 : 1 was found using almost 500 clinical samples (including nasopharyngeal swabs, sputum, saliva and fomites) from 75 patients. A ratio of approximately 10 000 : 1 was reported when using a more homogeneous virus that can be harvested from culture in a laboratory setting [82], in line with other studies [91]. Therefore, roughly assuming an RNA:PFU ratio of 10 000 : 1, air concentrations of 1 × 10^−3^ [73–75] gene copies per cm^3^ of sampled air would correspond to $1\times 10^{-7}\;{\mathrm{PFU}\;\;\mathrm{per}\;\;\mathrm{cm}}^{3}$ of air (or one PFU in ten cubic metres of air). Measurements of viral prevalence in indoor air include many variables depending on the situation. To model viral exhalations, it is preferred to use empirical data from direct measurements of viruses contained in exhaled air, or data of viral load contained in the respiratory tract fluid that is exhaled.

### 3.2. Prevalence of SARS-CoV-2 in air directly exhaled by infected persons

SARS-CoV-2 RNA has been detected in exhaled breath condensate (EBC), where participants’ exhaled breath is cooled and its contents are condensed into solution for analysis, without resolving the exhaled particle size distribution [74,92–95]. Some studies report that concentrations in excess of approximately $10^{-1}\;{\mathrm{gene}\;\mathrm{copies}\;\mathrm{per}\;\mathrm{cm}}^{3}$ of exhaled breath are possible, calculated based on their PCR results for EBC and the volume of air sampled [92,93]. Recent studies use a sampling apparatus which separates bioaerosols into ‘coarse’ (greater than 5 μm) and ‘fine’ (less than or equal to 5 μm) fractions to compare exhalations from breathing, speaking and coughing [96] or assess the performance of masks [97] for the amount of SARS-CoV-2 exhaled. These studies report significantly lower RNA exhalation rates than Ma *et al.* [92] reported for EBC. More data on direct measurements of exhalations are needed to provide more confidence in models of virus exhalations; however, these studies provide insights to quantify virus exhalaton rates [96,97].

Studies have not yet attempted to quantify indoor air samples relative to exhaled breath samples for the same participants, and comparisons between studies are subject to variabilities in viral load of patient, variant type, room air ventilation rates (and designed versus actual ventilation rates), variance in expiration rates based on patient (e.g. patient coughing vs breathing). A value of $10^{-3}\;{\mathrm{gene}\;\mathrm{copies}\;\mathrm{per}\;\mathrm{cm}}^{3}$ for room air [73–75] and $10^{-1}\;{\mathrm{gene}\;\mathrm{copies}\;\mathrm{per}\;\mathrm{cm}}^{3}$ for exhaled breath [92,93] would suggest a reasonable dilution ratio of 100, but this relation may be coincidental and more systematic sampling is required.

### 3.3. Prevalence of pathogens in respiratory fluid

Caution must be exercised if estimating viral load from samples of fluid extracted directly from the respiratory tract (e.g. nasopharyngeal swabs). Aerosols are plausibly generated from small airways [98,99], airway walls [100], larynx [99], and mucosalivary fluid from the mouth [27,101] by a range of mechanisms. Measurements of viral load in respiratory fluid span several orders of magnitude, they change over the course of the disease and can be different depending on the source of respiratory fluid [76,78,102].

To date, many studies have assumed a constant concentration of viruses in the fluid that composes the exhaled particles across the continuum of particle sizes [37] to assess relative risk rather than absolute risk of disease transmission associated with the modelled scenarios. Given that assumption, considering the cumulative volume fractions in figure 4 show the vast majority of respiratory fluid by volume is in the oral mode, it is expected that the vast majority of viral RNA detected would be found in the oral mode. However, recent data from the studies discussed in §3.2 question this assumption. Coleman *et al.* [96] reported from direct measurements of breathing, speaking and coughing that 85% of the detected RNA copies were found in the fine (less than or equal to 5 μm) aerosol fraction compared with the coarse (greater than 5 μm) aerosol fraction. Comparable results, where similar or more viral RNA is found in the fine aerosol mode, have been found for influenza [2,103,104], and these results have substantial implications for the relative importance of short- versus long-range transmission. However, the viral RNA possibly carried by the largest droplets may not be detected if they, for example, drop into the walls of the cone of the Gesundheit-II apparatus used in Coleman *et al.* [96] and are not retrieved. Cheng *et al.* [105] discussed the discrepancy between measurements of viral exhalations with other measurements of aerosol/droplet volumes as a function of particle size citing a possible gradient in viral load throughout the respiratory tract.

In light of this recent evidence, in electronic supplementary material, S3, we propose a method for scaling a viral load in the B and L modes relative to the O mode of the exhaled particles so that researchers can test their models in the limit where viral load in fine aerosols is significantly higher. We present this in general terms; such numerical values can be updated as more evidence becomes available. Taking the measurements from Coleman *et al.* [96], where 85% of the viral load to be in the particles with diameter less than 5 μm, we calculate that the viral load in the B and L modes would need to be 6 × 10^5^ times higher than the viral load in the O mode.

There is a critical need to improve the empirical data for the viral load in different particle sizes. Evidence from swab samples reported by Tu *et al.* [106] showed tongue swabs, perhaps representing the oral mode, contained generally lower viral RNA loads than NP swabs, perhaps representing the B and L models, though by 1–2 orders of magnitude, not 4–5.

#### 3.3.1. Correcting conversions for volumes of respiratory fluid

We do not recommend directly using clinical data of gene copies per ml reported for swab samples that have been diluted into another fluid. For example, nasopharyngeal swab samples are submerged and transported in viral transport media (typically in 3 ml of transport media) [78]. Subsequent measurements of viral RNA by PCR could be reported in gene copies per ml of transport media or gene copies per swab. However, the exact volume of respiratory fluid sampled on a given swab is unknown. While this is roughly of order 0.1 ml, the volume collected depends on the type of swab, practitioner and properties of the fluid. The dilution correction is therefore not well known and furthermore elution of viruses from the swab may be incomplete [107]. More discussion may be found in Roque *et al.* [108] which points out that if the average NP swab collects and releases 50 μl of nasal secretions and stores 3 ml of transport media, the original sample is diluted 60 : 1. Then, volumes extracted from the total solution for analyses by PCR must be correctly accounted for. These conversions may be estimated for modelling purposes; however, it must again be noted that the viral load may be different in different regions of the respiratory tract.

### 3.4. Experimental data needed for estimation of viral loads in aerosols and droplets

There are significant complexities of gathering experimental data relevant to disease transmission. Considering only aerosol sampling, it is difficult to gather size-resolved measurements of viral load in a controlled manner. For particle diameters larger than approximately 10 μm, competing transport phenomena (e.g. convection, gravity, inertial impaction) affect sampling, which may introduce bias in the reported results. Depending on the bioaerosol sampling method, the range of particle sizes sampled must be carefully considered. For example, the smallest particles less than 0.3 μm are inefficiently captured in a BioSampler [109]. Furthermore, for there to be an infection risk, the pathogen must be viable at the time of exhalation and must survive in the exhaled aerosol particles or droplets. The survival of viruses and bacteria in aerosols and droplets is highly dependent on the environmental conditions, such as the RH, temperature and exposure to light [41,56,70]. Therefore, it is critical that both gene copies and attempts to culture the virus are reported in measurements along with resolution of viral load as a function of particle size.

Additionally, more measurements of exhaled particle size distributions are needed. Specifically, since the particle sizes emitted vary by several orders of magnitude (approx. 0.01–1000 μm), more data are needed from instruments which complement one another to capture the entire size ranges of aerosols and droplets for the same exhalatory activities [110]. These data which are available in a controlled setting are critical to reconcile with viral exhalation rates described above, which are arguably more difficult to gather. By combining data of viral exhalations and aerosol/droplet exhalations, more accurate assessments of relative risk of different modes of transmission in specific scenarios are possible.

## 4. Summary and recommendations

There is rapid advancement in approaches to modelling the fate of exhaled particles in different environments. As these modelling approaches evolve, it is important that each model implementation can be verified by comparison with others, and that any differences in results can be attributed to incomplete specification or alternative implementation of the physics. With this paper, we provide a consolidated set of parameters for exhalation of particles that are appropriate to be used by a range of modelling approaches as the basis for model inter-comparison and benchmarking. This paper is not intended as a formal systematic review and therefore it is likely that there is some selection bias in our identification of literature. While we applied expert judgement to evaluate the merits of different papers, paying close attention to methods and strength of evidence, we only reviewed papers written in English and did not formally apply pre-defined quality criteria to evaluate the strengths and weaknesses of different studies.

In reporting results, details of all physical and mathematical models should be provided along with a description of the modelled scenario including a diagram, dimensions, and all boundary conditions. It is necessary to resolve particle transport (and deposition) as a function of particle diameter, therefore distributions of both number concentrations and volume concentrations (as shown in figure 4) should be reported as a function of time and spatial location relative to the particle source (e.g. in vertical and horizontal cross-sections). By reporting distributions of particles by volume, models for viral load within each particle may be readily applied to model virus dispersal and deposition, allowing relative assessments of risk relative rather than absolute assessments of risk.

We note that there is significant person-to-person variability in exhaled air flows, exhaled particle distributions and composition, and, perhaps most significantly, in viral load. The evidence base for the statistical distribution of these parameters within the population is incomplete; different studies typically have small sample sizes and are not often directly comparable, for example due to different vocal activities and measurement methods. Thus, there is insufficient evidence to quantify the modal, mean or median parameter values within the population. We therefore strongly encourage modellers to account for the sensitivity of their results to these uncertainties: exhaled air flow variability could be quantified using distributions of body height and weight [20,24]; a number of different measured exhaled aerosol distributions are presented in electronic supplementary material, S3; different representations of respiratory droplet composition could be used [38,42,111]; and the large range in viral load discussed in §3 must be accounted for in any attempt to quantify the absolute risk of transmission.

While this paper focuses on defining a set of terms for model benchmarking, modellers may benefit from an additional set of terms that can be used to evaluate how model outputs change with respect to variations in the source terms. For this purpose, we provide an additional set of source terms in table S2 in electronic supplementary material (S4) with different parameter values that represent variations in exhalation for the average British male and female and variations in particle composition and environmental conditions that are consistent with the ranges used in the modelling study of de Oliveira *et al.* [11].

There remain a significant number of outstanding questions related to airborne transmission of pathogens. Modelling the fate of exhaled particles, when implemented with careful verification of methods and experimental validation, can help to understand possible transmission pathways and inform efforts to mitigate transmission.

## Data Availability

The data are provided in electronic supplementary material [112].

## Footnotes

1 Studies commonly report gene copies of a target gene (e.g. N gene of SARS-CoV-2) which are converted from cycle thresholds (Ct) by calibration for a given PCR system. Cycle thresholds represent the number of amplification cycles used to detect a target gene by PCR, where lower Ct values correspond to higher numbers of gene copies. Since Ct are platform-dependent, it is preferred to compare among studies using gene copies determined by a standard calibration procedure.
